# Cys Site-Directed Mutagenesis of the Human SLC1A5 (ASCT2) Transporter: Structure/Function Relationships and Crucial Role of Cys467 for Redox Sensing and Glutamine Transport

**DOI:** 10.3390/ijms19030648

**Published:** 2018-02-25

**Authors:** Mariafrancesca Scalise, Lorena Pochini, Lara Console, Gilda Pappacoda, Piero Pingitore, Kristina Hedfalk, Cesare Indiveri

**Affiliations:** 1 Department DiBEST (Biologia, Ecologia, Scienze della Terra) Unit of Biochemistry and Molecular Biotechnology, University of Calabria, Via P. Bucci 4C, 87036 Arcavacata di Rende, Italy; mariafrancesca.scalise@unical.it (M.S.); lorena.pochini@unical.it (L.P.); console@hotmail.it (L.C.); gilda.pappacoda@gmail.com (G.P.); 2 Department of Molecular and Clinical Medicine, Wallenberg Laboratory, Sahlgrenska Academy, University of Gothenburg, 413 45 Gothenburg, Sweden; piero.pingitore@wlab.gu.se; 3 Department of Chemistry and Molecular Biology, University of Gothenburg, P.O. Box 462, SE-405 30 Göteborg, Sweden; kristina.hedfalk@gu.se; 4 CNR Institute of Biomembranes, Bioenergetics and Molecular Biotechnology, via Amendola 165/A, 70126 Bari, Italy

**Keywords:** amino acid, glutamine, transport, over-expression, site-directed mutagenesis, liposome

## Abstract

The human plasma membrane transporter ASCT2 is responsible for mediating Na- dependent antiport of neutral amino acids. New insights into structure/function relationships were unveiled by a combined approach of recombinant over-expression, site-directed mutagenesis, transport assays in proteoliposomes and bioinformatics. WT and Cys mutants of hASCT2 were produced in *P. pastoris* and purified for functional assay. The reactivity towards SH reducing and oxidizing agents of WT protein was investigated and opposite effects were revealed; transport activity increased upon treatment with the Cys reducing agent DTE, i.e., when Cys residues were in thiol (reduced) state. Methyl-Hg, which binds to SH groups, was able to inhibit WT and seven out of eight Cys to Ala mutants. On the contrary, C467A loses the sensitivity to both DTE activation and Methyl-Hg inhibition. The C467A mutant showed a Km for Gln one order of magnitude higher than that of WT. Moreover, the C467 residue is localized in the substrate binding region of the protein, as suggested by bioinformatics on the basis of the EAAT1 structure comparison. Taken together, the experimental data allowed identifying C467 residue as crucial for substrate binding and for transport activity modulation of hASCT2.

## 1. Introduction

ASCT2 (SLC1A5) is a neutral amino acid transporter of plasma membrane that belongs to the SLC1 family together with ASCT1 (SLC1A4) and five glutamate transporters (SLC1A1–3 and SLC1A5–6). Tissue distribution of ASCT2 is quite broad, being expressed in kidney, intestine, brain, lung, skeletal muscle, placenta and pancreas where ASCT2 is mainly involved in traffic of neutral amino acids [1]. Murine ASCT2 members have been firstly studied in different experimental models, i.e., cells and proteoliposomes [2,3]. In these studies, some important features of ASCT2 have been described such as the specificity towards amino acids and the mode of transport; in particular, Gln was revealed to be one of ASCT2 preferred substrates. The transport reaction is a sodium dependent antiport in which Gln is co-transported with Na^+^ in exchange with another neutral amino acid [2,3]. However, the murine ASCT2 proteins show large local dissimilarities with respect to the human ASCT2 [4]. Such a low homology between murine and human orthologues is not common for most membrane transporters, which are characterized by a quite high similarity [5]. Thus, murine ASCT2 are not straightforward models for the human counterpart. Therefore, the interest in deciphering the molecular mechanism and the regulation of the human orthologue of ASCT2 became very remarkable.

### 1.1. Relevance of ASCT2 to Metabolism

The relevance of the hASCT2 knowledge was further increased by the large number of reports describing over-expression of this protein in human cancers [6,7]. This is a typical hallmark of cancer cells which are, indeed, “Gln addicted” [6]. In this scenario, ASCT2 is considered as a key player in providing cancer cells with Gln, whose carbon skeleton is then used for ATP production. The most plausible mechanism underlying this phenomenon is that Gln is exchanged with smaller amino acids such as Ser or Thr triggering a net accumulation of one or two carbon atom(s) useful for energy production in mitochondria by a truncated form of TCA in which ATP is synthesized at substrate level [8].

### 1.2. Functional and Kinetic Characterization of ASCT2

**Figure 1:**
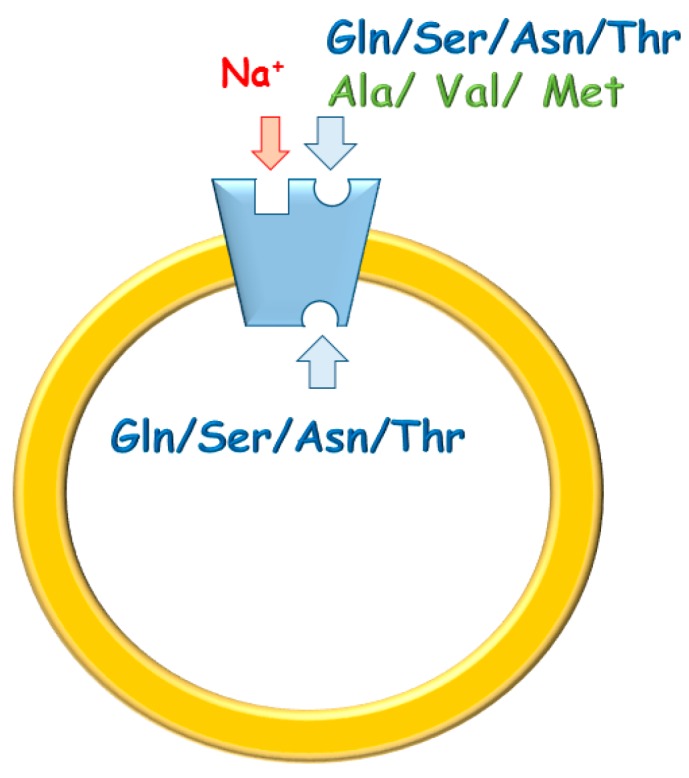
Sketch of hASCT2 and its transport mode. The protein is inserted asimmetrically in the native membrane and catalyzes a three substrates antiport. In blue, substrates bidirectinally transported; in green, substrates only inwardly transported. In red, Na^+^ site that takes part in the transport cycle.

Thus, the knowledge on hASCT2 function was recently enlarged from the animal to the human transporter obtained by recombinant expression in yeast, subsequent purification and reconstitution in proteoliposomes [4,9,10]. In these studies, it has been definitively demonstrated that the transport reaction catalyzed by the hASCT2 can be assimilated to a three-substrates reaction occurring with a random simultaneous mechanism [9]. Functional and kinetic asymmetry of hASCT2 has been described, which reflect the physiological role of the transporter in regulating extra/intracellular amino acid pools (Figure 1) [9]. The electrogenic property of the three-substrate reaction has been finally assessed solving previous controversy deriving from studies conducted on the rat protein [11]. Moreover, intracellular sodium activates the transport acting as an allosteric modulator; this property is shared with the rASCT2 [11]. More recently, it was shown that Cys, historically considered as a major substrate, is not transported by human ASCT2 but behaves as a regulator, tuning unidirectional efflux activity. Interestingly, this action does not occur through formation of mixed disulfide with thiol residues of the protein [10].

### 1.3. Regulatory Properties of ASCT2

The trafficking of hASCT2 to the plasma membrane has been also studied by site-directed mutagenesis revealing that N-glycosyl moieties linked to N163 and N212 are responsible for routing hASCT2 to plasma membrane without affecting intrinsic transport activity [12]. Finally, it has been described that the scaffold protein PDZK1 binds to the C-ter of hASCT2: however, the function of this interaction is still not assessed [4]. An intriguing characteristic of the hASCT2 is that the number of Cys residues is halved compared to the rat counterpart [4]. Since it is well known that Cys residues are involved in the control of redox state of transporters and in several types of Post Translational Modifications [13,14,15,16,17], we started to investigate the role of hASCT2 Cys residues. This interest has a double rationale: on the one hand, gaining further insights on structure/function relationships of hASCT2, on the other hand, exploiting Cys residues as a target of covalent inhibitors with potential application as anticancer drugs. Interestingly, compounds reacting with a Cys residue located in the substrate binding were designed and revealed to be potent and specific inhibitors of hLAT1 by in vitro and ex vivo experimental models [18].

## 2. Results

### 2.1. Effect of Reducing Agents on Wild Type hASCT2

**Figure 2:**
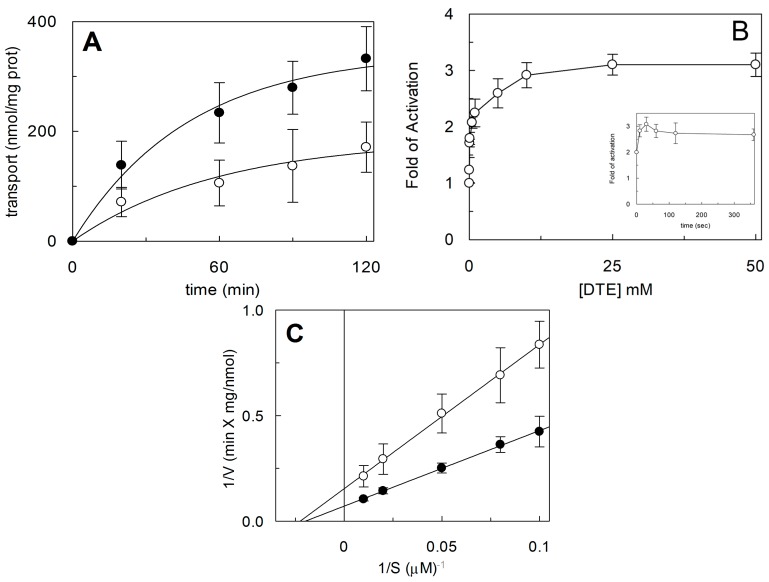
Effect of DTE on hASCT2 transport activity. The reconstitution was performed as described in Section 4.5. (**A**) Transport was started by adding 50 μM [^3^H]Gln and 50 mM external Na-gluconate at time zero to proteoliposomes containing 10 mM Gln in the presence (●) or absence (○) of 10 mM DTE. The transport reaction was stopped at the indicated times, as described in Section 4.6. (**B**) Transport was started by adding 50 μM [^3^H]Gln and 50 mM external Na-gluconate at time zero to proteoliposomes reconstituted with hASCT2 and containing 10 mM Gln upon 30 s of incubation with the indicated concentrations of extraliposomal DTE. In the inset, the dependence on incubation time is reported using 10 mM DTE. Transport activity was shown as fold of activation, in the presence of DTE, with respect to absence of DTE. The transport reaction was stopped after 30 min as described in Section 4.6. (**C**) Kinetic analysis of hASCT2 transport activity. Transport rate was measured adding [^3^H]Gln at the indicated concentrations and 50 mM Na-gluconate, in the presence (●) or absence (○) of 10 mM DTE, to proteoliposomes containing 10 mM Gln. The transport reaction was stopped after 15 min as described in Section 4.6. Data were plotted according to Lineweaver-Burk as reciprocal transport rate vs reciprocal Gln concentration. (**A**–**C**) Results are means ± S.D. from three experiments.

**Figure 3:**
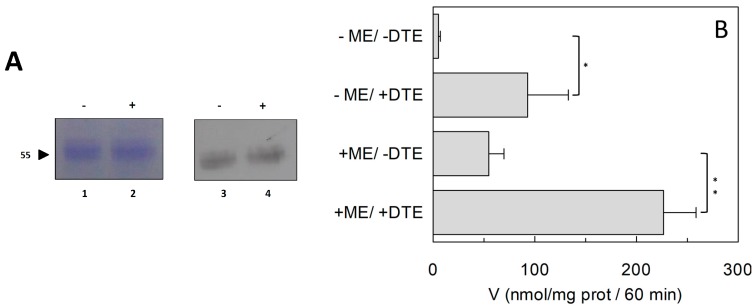
Effect of reducing agent on purification and activity of hASCT2. (**A**) Blu Coomassie Staining (lanes 1 and 2) and Western blot analysis (lanes 3 and 4) of purified hASCT2. Protein was prepared in the absence (lanes 1 and 3) or presence (lanes 2 and 4) of β-mercaptoethanol in purification buffers. Samples were analyzed on SDS–PAGE 12% and transferred onto nitrocellulose membrane. Immuno-detection was performed using anti-His as described in Section 4.4. (**B**) The reconstitution was performed as described in Section 4.5 using protein prepared in the presence or absence of β-mercaptoethanol as in Figure 2A. Transport was started by adding 50 μM [^3^H]Gln and 50 mM external Na-gluconate, in the presence or absence of 10 mM DTE, at time zero to proteoliposomes containing 10 mM Gln. The transport reaction was stopped after 60 min, as described in Section 4.6. Results are means ± S.D. from three experiments. Student’s two tailed unpaired *t*-test was performed on the sample without DTE in the transport buffer; *p* values were symbolized as follows: * *p* < 0.05; ** *p* < 0.01.

**Figure 4:**
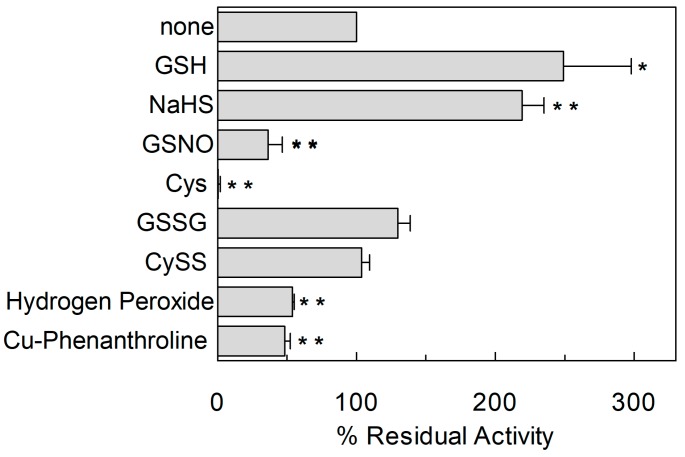
Effect of reducing and oxidizing agents on hASCT2 transport activity. The reconstitution was performed as described in Section 4.5. Transport was started by adding 50 μM [^3^H]Gln and 50 mM external Na-gluconate at time zero to proteoliposomes containing 10 mM Gln in the presence of 5 mM GSH, 100 µM NaHS (H_2_S donor), 1 mM GSNO (NO donor), 1 mM Cys, 5 mM GSSG, 5 mM CySS, 1 mM Hydrogen Peroxide or 100 µM Cu-Phenanthroline. Transport activity was calculated as percent of residual activity with respect to condition without any addition. The transport reaction was stopped after 30 min, as described in Section 4.6. Results are means ± S.D. from three experiments. Student’s two tailed unpaired *t*-test was performed on the sample without any addition (none) in the transport buffer; *p* values were symbolized as follows: * *p* < 0.05; ** *p* < 0.01.

To investigate the influence of the redox state of Cys on transport function, the thiol residues of the protein were reduced by DTE and the Na^+^-dependent [^3^H]Gln_ex_/Gln_in_ antiport was measured in proteoliposomes in which the recombinant ASCT2 is inserted with the same orientation as in the native membrane (Figure 1) [4]. As shown in Figure 2, DTE stimulated the time dependent accumulation of Gln in proteoliposomes. Initial transport rates were calculated as k limit from the first order rate equation used to plot the experimental data. The rate of 3.2 ± 1.2 nmol·min^−1^·mg·protein^−1^ of the control, increased up to 7.3 ± 1.8 nmol·min^−1^·mg·protein^−1^ upon DTE addition (Figure 2A). Transport at equilibrium was almost doubled from 171 nmol·mg·protein^−1^ to 332 nmol·mg·protein^−1^. The effect of DTE was concentration and time dependent as shown in Figure 2B with maximal activity at 10 mM DTE after 30 s incubation (Figure 2B, inset). Kinetic analysis was performed under both reducing and non-reducing conditions, i.e., in the presence or absence of DTE, respectively. In these experiments, transport rate was measured at increasing external [^3^H]Gln concentrations and data were plotted according to Lineweaver-Burk equation (Figure 2C). The Vmax increased from 6.75 ± 2.27 to 14.7 ± 2.78 nmol·min^−1^·mg·protein^−1^. On the contrary, the external Km value for Gln was unaffected and it was similar to that previously reported [9]. The observed results indicated that a fraction of the protein was inactive under oxidized conditions and, hence, the inactive form of the protein was associated to the presence of disulfide(s). To gain further insights into the described effects, a more oxidized protein preparation was used for the transport measurements. The oxidized protein was obtained excluding the reducing agent β-mercaptoethanol from the purification buffers (see Section 4.4). In this condition, possibly a higher number of disulfides is present. Indeed, the transport activity of ASCT2 was negligible even though the efficiency of purification was not affected, as demonstrated by the unchanged amount of purified protein detected by both Blue Coomassie staining and WB analysis with a specific antibody (Figure 3A). Interestingly, the following addition of DTE to the transport buffer led to a terrific recovery of activity (Figure 3B). This phenomenon varied a lot among the different protein preparations; however, after removal of β-mercaptoethanol and subsequent treatment with DTE, the transporter did not reach the full activity, i.e., that obtained under the normal conditions of purification (Figure 3B). This unpredictable behavior could be ascribed to the random formation of disulfides that does not allow a proper folding of the protein and a correct insertion in the proteoliposome membrane. Therefore, all the following experiments were performed under purification conditions of Figure 2, i.e., using protein(s) prepared with β-mercaptoethanol in purification buffers (see Section 4.4). Moreover, if the same concentration of β-mercaptoethanol was maintained during all the steps, from purification to transport assay, the transport activity corresponded to that obtained after addition of DTE to the protein purified in the presence of β-mercaptoethanol. All data indicated that the presence of β-mercaptoethanol during purification guaranteed a correct protein folding and insertion in the membrane. After this step, the protein undergoes to spontaneous oxidation that can be fully reversed by the reducing chemical reagent DTE. Then, physiological reducing agents GSH, H_2_S, Cys, physiological and not physiological oxidizing agents such as GSSG, Cystine, H_2_O_2_, CuPhe as well as the free radical Nitric Oxide (NO) able to interact with Cys residues, were tested for their capacity to rescue the hASCT2 function after spontaneous oxidation (Figure 4). The gas transmitters H_2_S and NO were generated by adding 0.1 mM NaHS or 1 mM GSNO to the samples, respectively. It has to be stressed that the actual concentration of the released gases is much lower with respect to the indicated concentrations of the donors and, hence, closer to physio/pathological concentrations [13,15]. In line with DTE effects, GSH and H_2_S stimulated transport activity of hASCT2 to a similar extent than DTE. While Cys strongly inhibited the transport; in this case, inhibition occurred by a mechanism not involving S-S/SH inter-conversion, as previously demonstrated [10]. GSSG and Cystine did not elicit any effects at the tested concentration; while NO, H_2_O_2_ and Cu-Phenanthroline inhibited transport by more than 50%.

### 2.2. Homology Structural Model of hASCT2 and Effects of SH-Reagents on WT

**Figure 5:**
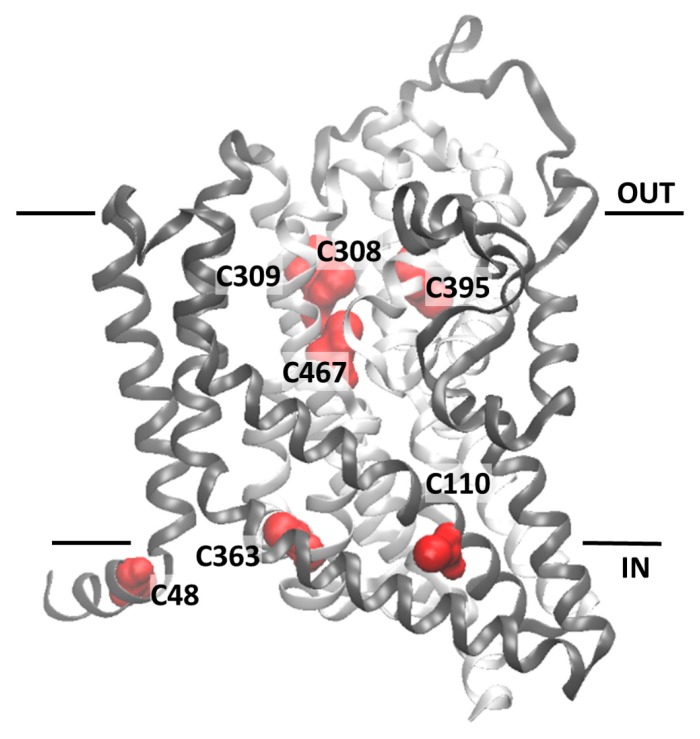
Homology structural model of the hASCT2. The model was obtained using the crystallographic structure of EAAT1 (PDB 5LLU) as template (Section 4.5). Panel shows the ribbon diagram highlighting 7 out of the 8 Cys residues of the human ASCT2 which are depicted in red; C308, C309, C395 and C467 are located in the core of the protein named “elevator” which is represented in light gray. C48, C110 and C363 are located in external α-helices depicted in dark gray. The homology model was represented using the molecular visualization program VMD. Models were generated by different types of software. The one depicted in the figure, from Swiss Model, represents the best compromise between the sequence coverage (83%) and number of amino acids (95%) falling in the favored region of the Ramachandran plot.

**Figure 6:**
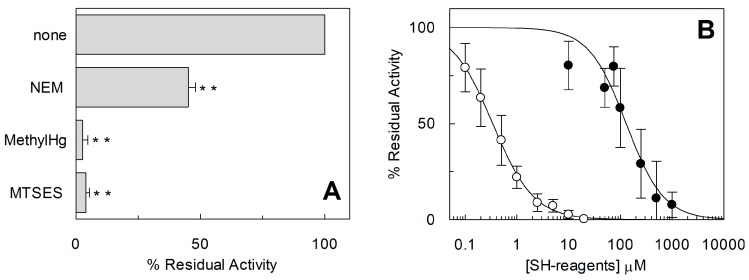
Effect of SH-reagents on hASCT2 transport activity. The reconstitution was performed as described in Section 4.5. (**A**) Transport was started by adding 50 μM [^3^H]Gln and 50 mM external Na-gluconate at time zero to proteoliposomes containing 10 mM Gln in the presence of 1 mM NEM, 10 µM Methyl-Hg or 1mM MTSES. Transport activity was calculated as percent of residual activity with respect to condition without any addition. (**B**) Dose–response curves for the inhibition of the hASCT2 in proteoliposomes by MTSES (●) and Methyl-Hg (○). Transport was measured adding 50 mM Na-gluconate and 50 µM [^3^H]Gln to proteoliposomes containing 10 mM Gln in the presence of indicated concentrations of SH-reagents. Transport activity was calculated as percent of residual activity with respect to condition without any addition. The transport reaction was stopped after 30 min, as described in Section 4.6. (**A**,**B**) Results are means ± S.D. from three experiments. Student’s two tailed unpaired *t*-test was performed on the sample without any addition (none) in the transport buffer; *p* value was symbolized as follows: ** *p* < 0.01.

The homology structural model of hASCT2 was built using as a template the recently published crystal structure of hEAAT1 (SLC1A1, PDB 5LLU) [19] (Figure 5). However, the amino acid residues 1–41, including C39, could not be modeled because no corresponding residues were present in the template. All the other Cys residues are highlighted in the structural model (Figure 5). Five Cys residues are localized in the elevator moiety of the protein, which is the supposed mobile domain allowing substrate transfer from one side to the other of the membrane [20]: C308, C309, C363, C395 and C467. While C48 and C110 are on the external α-helices protruding towards the membrane forming the fix domain of the protein. At this stage, we can only speculate that C39, which is not reported in the model, occupies an external stretch. To gain information on the possible involvement of the thiol moiety on the protein function, hydrophobic (membrane permeable) and hydrophilic (membrane impermeable) SH reagents were used and their effect on the transport activity was evaluated (Figure 6). The membrane permeable NEM led to only 50% inhibition, while Methyl-Hg and the hydrophilic MTSES led to complete inhibition (Figure 6A). Then, dose-response analyses were conducted on Methyl-Hg and MTSES from which IC_50_ value of 0.33 ± 0.02 and 129 ± 21 µM (Figure 6B) were derived, respectively. These data indicated a much higher affinity of the transporter for Methyl-Hg than for MTSES. The higher inhibitory effect of MTSES with respect to NEM could be explained by the presence of the positively charged residues in the vicinity of C467 that facilitate the interaction with MTSES.

### 2.3. Site-Directed Mutagenesis and Transport Activity of Cys-Ala Mutants of hASCT2

**Figure 7:**
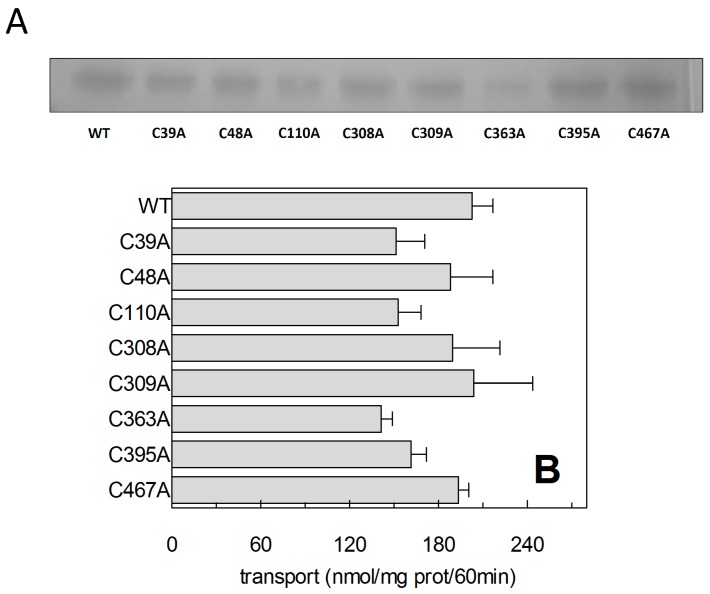
Recombinant hASCT2-6His expression. (**A**) WT ASCT2 and indicated Cys-Ala mutants were produced and purified as described in Section 4.3. Purified proteins were separated by 12% SDS-PAGE and stained with Coomassie Blue. (**B**) Transport activity of WT and Cys-Ala mutants was evaluated in proteoliposomes. The reconstitution was performed as described in Section 4.5 and transport was started by adding 50 μM [^3^H]Gln and 50 mM external Na-gluconate at time zero to proteoliposomes containing 10 mM Gln. The transport reaction was stopped after 60 min, as described in Section 4.6. Results are means ± S.D. from three experiments.

**Figure 8:**
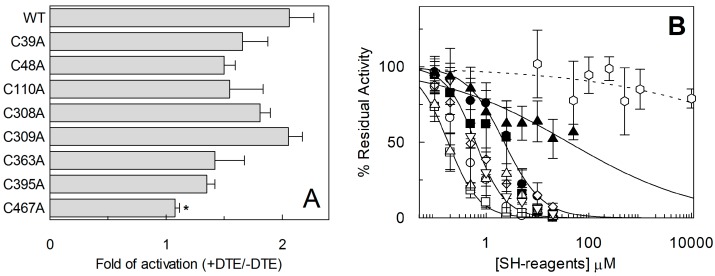
Measurement of transport activity of WT ASCT2 and Cys-Ala mutants. The reconstitution was performed as described in Section 4.5. (**A**) Transport was started by adding 50 μM [^3^H]Gln and 50 mM external Na-gluconate at time zero, in the presence and absence of 10 mM DTE, to proteoliposomes containing 10 mM Gln. The transport reaction was stopped after 60 min, as described in Section 4.6. Transport activity was shown as fold of activation, in the presence of DTE, with respect to absence of DTE. Student’s two tailed unpaired *t*-test was performed on the WT sample; *p* value was symbolized as follows: * *p* < 0.05. (**B**) Dose–response curves for the inhibition of the hASCT2 Cys-Ala mutants in proteoliposomes by Methyl-Hg. Transport was measured adding 50 µM [^3^H]Gln and 50 mM Na-gluconate to proteoliposomes containing 10 mM Gln in the presence of indicated concentrations of Methyl-Hg. Transport activity was calculated as percent of residual activity with respect to condition without any addition: C39A (○), C48A (●), C110A (□), C308A (◊), C309A (▪), C363A (X), C467A (χ). The transport reaction was stopped after 30 min, as described in Section 4.6. Measured IC_50_ were 0.4 ± 0.1, 2.2 ± 0.8, 0.18 ± 0.05, 0.64 ± 0.2, 1.38 ± 0.5, 0.62 ± 0.2 and 40 ± 0.9 µM for C39A, C48A, C110A, C308A, C309A, C363A and C467A, respectively. In dashed line, dose-response analysis of C467A with indicated concentrations of MTSES (Φ); IC_50_ value not determined. (**A**,**B**) Results are means ± S.D. from three experiments.

The described thiol reagents could be suitable for identifying the critical Cys residue(s) of the protein. Thus, site directed mutagenesis of hASCT2 was undertaken and substitution of each of the eight Cys to Ala was performed (see Section 4.2 and Figure 7A). Mutants were firstly tested in proteoliposomes revealing that all mutants were functional (Figure 7B): C39A, C363A and C395A showed the lowest activity that, anyway, was more than 75% of the WT. Surprisingly, *P. pastoris* cells were refractory to express the mutant C363A (Figure 7A). To obtain an appreciable level of expression, the growth conditions were changed with respect to those of the WT protein and the other mutants (see Section 4.2). To identify the major Cys residue(s) responsible for activation/inhibition of the transporter, the effect of DTE and Methyl-Hg was tested on each mutant (Figure 8). Interestingly, all mutants showed a more or less pronounced stimulation by DTE with the only exception of C467A, which was insensitive to reducing agent (Figure 8A). Dose-response analysis performed with Methyl-Hg revealed that C467A mostly lost sensitivity to the reagent with a measured IC_50_ more than two orders of magnitude higher than that of WT, i.e., about 40 µM (Figure 8B). While, the calculated IC_50_ values for other mutants was not very far from that of WT (see legend to Figure 8B). These results were in favor of a major role of C467 in defining the relationships between the Cys residues of the protein and the transport function. In line with this finding, the C467A mutant completely lost the reactivity towards the reagent MTSES (Figure 8B). Noteworthy, according to the model of Figure 5, C467 is located in the core of the protein.

### 2.4. Functional and Kinetic Characterization of C467A Mutant

**Figure 9:**
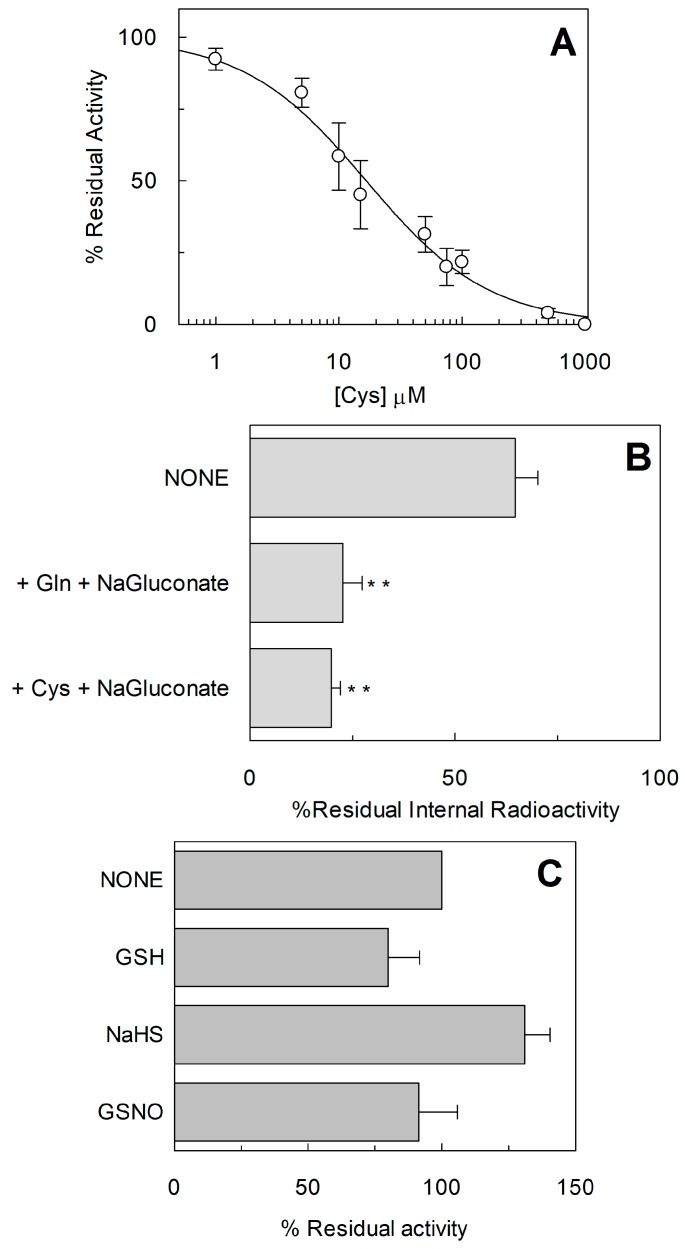
Functional characterization of C467A. (**A**) Dose–response curve for the inhibition of the hASCT2 C467A in proteoliposomes by Cys. The reconstitution was performed as described in Section 4.5 and transport was measured adding 50 mM Na-gluconate and 50 µM [^3^H]Gln to proteoliposomes containing 10 mM Gln in the presence of indicated concentrations of Cys. Transport activity was calculated as percent of residual activity with respect to condition without any addition. (**B**) Efflux of Gln from proteoliposomes reconstituted with C467A hASCT2 mutant. The reconstitution was performed as described in Section 4.5 and uptake of 50 µM [^3^H]Gln in proteoliposomes was performed in the presence of 50 mM Na-Gluconate. After accumulation of [^3^H]Gln for 120 min, [^3^H]Gln efflux was measured in the presence of 50 mM Na-gluconate and 1 mM Gln or 1 mM Cys. After complete Gln efflux, aliquots of proteoliposomes were passed through Sephadex G75 column to remove external substrates. Percent of intraliposomal residual radioactivity is reported compared to control. Student’s two tailed unpaired *t*-test was performed on the WT sample; *p* value was symbolized as follows: ** *p* < 0.01. (**C**) The reconstitution was performed as described in Section 4.5 and transport was measured adding 50 mM Na-gluconate and 50 µM [^3^H]Gln to proteoliposomes containing 10 mM Gln in the presence of 5 mM GSH, 100 µM NaHS (H_2_S donor) or 1 mM GSNO (NO donor). Transport was calculated as percent of residual activity with respect to condition without any addition. (**A**–**C**) Results are means ± S.D. from three experiments.

**Figure 10:**
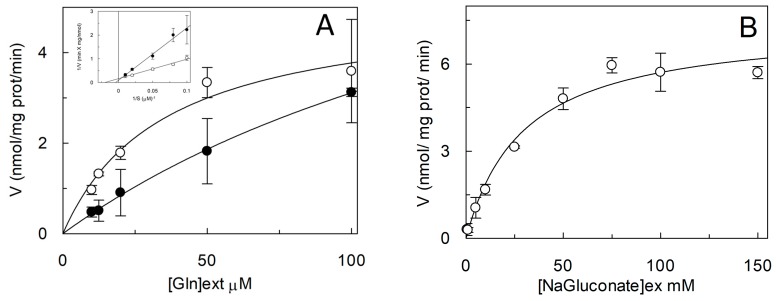
Kinetics of C467A mutant. (**A**) The reconstitution of WT (○) and C467A (●) was performed as described in Section 4.5 and transport rate was measured adding 50 mM Na-gluconate and [^3^H]Gln at the indicated concentrations to proteoliposomes containing 10 mM Gln. Transport was stopped after 15 min as described in Section 4.6. Data are plotted according to Michaelis- Menten equation, in the inset the same data are plotted according to Lineweaver–Burk as reciprocal transport rate vs reciprocal Gln concentration. (**B**) Kinetic analysis of C467A mutant. Transport rate was measured adding 50 µM [^3^H]Gln and the indicated concentration of Na-Gluconate to proteoliposomes containing 10 mM Gln. Data are plotted according to Michaelis–Menten equation. (**A**,**B**) Results are means ± S.D. from three experiments.

**Figure 11:**
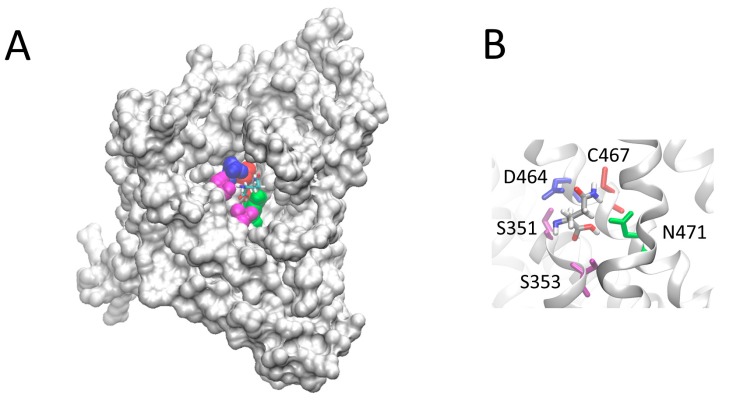
View of the hASCT2 residues, which interact with glutamine. In (**A**), the space-filled diagram highlighting the lateral view of the residues involved in interactions with the substrate. In (**B**), an enlarged view of the residue interacting with the Gln. The highlighted amino acids are depicted in a ball and stick representation. C467 is depicted in red, D456 in blue, S351 and S353 in magenta, N463 in green. The 3D model has been represented using the molecular visualization program VMD. The position of Gln within the protein has been optimized using the software ArgusLab (M.A. Thompson, ArgusLab 4.0.1 Planaria Software LLC, Seattle, WA, USA, (2004) available online: http://www.arguslab.com).

As stated in the introduction, it was described that Cys exerted some modulation of the hASCT2 transport function both on the native (intact cells) and on the recombinant protein independently from interaction with thiol groups of the protein [10]. Thus, the effect of Cys was also evaluated on the uptake of Gln mediated by C467A mutant (Figure 9A). Interestingly and in line with previous results, from the dose-response analysis, an IC_50_ of 16.6 ± 1.62 µM was measured. Moreover, Cys was also able to induce potent Gln efflux from reconstituted proteoliposomes (Figure 9B). These effects overlapped that previously described for WT [10]. To gain further insights on the possible role of C467 in the modulation of the redox state of hASCT2, its reactivity towards physiological SH reagents was tested (Figure 9C): these compounds were not able to stimulate nor to inhibit C467A transport activity, differently from their effect on WT (Figure 4). To investigate the possible role of C467 in the transport cycle, kinetic parameters were also measured: external Km for Gln was increased up to 202 ± 33 µM, i.e., about one order of magnitude that of WT (35 ± 10 µM; Figure 10A). On the contrary, Km for Na^+^ was 29.3 ± 1.41 mM and it was unchanged with respect to that of WT previously measured (Figure 10B) [9]. These results suggested that the C467 might play a role in binding and transport of the substrate. In line with these data, the Figure 11 shows the C467 is surrounded by four residues, D464, S351, S353, N471 which correspond to the residues of hEAAT1 involved in substrate binding and translocation [19].

## 3. Discussion

The structure/function relationships of hASCT2 were investigated by evaluating the effects of SH targeting reagents on the WT protein and on the Cys-Ala mutants constructed by site-directed mutagenesis and tested in proteoliposome-based assays. This combined experimental strategy disclosed some interesting aspects of hASCT2 substrate binding site and suggested possible mechanisms of regulating its function. The opposite effects exerted on transport activity by reducing or oxidizing agents correlated with the reduced (thiol) or oxidized (disulfide) state of Cys residue(s) of the protein. In other words, the S-S/SH interconversion of Cys residue(s) switches the protein from “OFF” to “ON” state. This is also in good agreement with the finding that the Vmax of transport, but not the Km, was affected by the reduced (SH) or oxidized (S-S) state of Cys residues.

Moreover, the unvaried Km can be explained by the presence of a mixed population of protein, made both by active (reduced) and inactive (oxidized) form. Thus, when protein is fully reduced is more active; on the contrary, when S-S is formed, protein is blocked in a less mobile conformation. The homology model, built on the basis of the structure of human EAAT1 recently solved [19], was used to evaluate the location and the exposure of the eight Cys residues of hASCT2. Noteworthy, SLC1 family members have a different number of Cys residues and the only one conserved among all proteins is that corresponding to C363 of hASCT2 [19]. This residue is located in a transmembrane segment called HP1b, that is part of the elevator portion of the protein (Figure 5), which may underlie a mechanism of transport shared also with the bacterial homologue GLTph [20]. Interestingly, data from site-directed mutagenesis highlighted that no relationships to both DTE activation and the Methyl-Hg inhibition could be ascribed to this residue, as well as to none of other six Cys-Ala mutants (Figure 8B). Therefore, the conservation of C363 can have a different explanation being related to the structural stability and/or folding of the protein, in line with the reluctance of *P. pastoris* to over-express the C363A mutant. In fact, after 24 h of induction of protein expression, the level of C363A mutant dropped down, suggesting a possible toxicity/aggregation of this mutant as already reviewed for other over-expressed proteins [21]. Noteworthy, C467A is the only mutant that nearly lost reactivity towards DTE, Methyl-Hg and MTSES. The same effects, even if at slightly different extent, were exerted by the physiological reducing agents GSH, the gas transmitter H_2_S and NO that, differently from the WT, were not able to modulate C467A transport activity. Concerning the role of C467 in disulfide formation, one or more Cys, besides C467, should be involved. From the present data, it is clear the disulfide counterparts are more than one, since no other mutant, apart C467, loses the sensitivity to reducing agents. The most plausible interpretation is that the intrinsic protein mobility allows different Cys SH residues coming in proximity of C467, with consequent formation of a disulfide. Thus, the substitution of one of these Cys residues does not alter the propensity to form disulfides except in the case of C467, which is the core residue. Moreover, from either previous [4,9] and present data, it could be argued that the Cys residues are not involved in ASCT2 oligomerization. These features are not merely structural but may have important outcomes in the explanation of the mechanism of transport of ASCT2. In fact, the C467 residue corresponds to the crucial R457 residue in EAAT1 and to the T459 of ASCT1 [19]. These differences may be explained in the light of substrate specificities of each of the mentioned transporters. In this respect, Arg can be the site responsible for interaction with negatively charged amino acid in EAATs, while can be replaced by other amino acids in the case of ASCTs. In fact, Cys and Thr are characterized by the presence of -SH or -OH functional group, respectively, which can give rise to non-ionic interactions with Gln or other neutral amino acids (Figure 11). In line with this hypothesis, a mutation of T459 of ASCT1 to Arg changed the substrate specificity of ASCT1 that became able to transport also Glu [22]. The role of C467 in coordinating neutral amino acids as substrates of the transporter is suggested by the much higher Km for Gln. However, this diminished affinity towards Gln does not interfere with the Km for Na^+^ that was unchanged meaning that Na^+^ binding should occur at another site. A further relevant aspect of C467A mutant is that its reactivity towards Cys was unchanged with respect to the WT, confirming that the regulatory role of Cys on ASCT2 transport activity does not occur at the level of the substrate binding site of the protein as previously suggested [10].

## 4. Materials and Methods

### 4.1. Materials

The *P. pastoris* wild type strain (X-33), the pPICZB vector, zeocin, Ni-NTA agarose resin were from Invitrogen; restriction endonucleases and other cloning reagents were from Fermentas; PD-10 columns, ECL plus, Hybond ECL membranes were from GE Healthcare; l-[^3^H]Gln was from Perkin Elmer; conjugated anti-His_6_ antibody, C_12_E_8_, Amberlite XAD-4, egg yolk phospholipids (3-sn-phosphatidylcoline from egg yolk), Sephadex G-75, l-Gln and all the other reagents were from Sigma-Aldrich.

### 4.2. Generation and Cloning of hASCT2 Mutants

The human ASCT2 gene was codon optimized for *P. pastoris* by GenScript and cloned in pPICZB expression vector as described in [9]. Seven out of the eight Cys residues were mutated to Ala by PCR overlap extension method [13,23] using the following primers and cloned with the same strategy used for WT:

C39A Forward GTGCTGCCGCAGGTGGATACGCTGGATCCAGAGATCAAGTCAGAAG
C39A Reverse CTTCTGACTTGATCTCTGGATCCAGCGTATCCACCTGCGGCAGCAC
C48A Forward GATCAAGTCAGAAGAGCTTTGAGAGCTAACTTGC
C48A Reverse GCAAGTTAGCTCTCAAAGCTCTTCTGACTTGATC
C110A Forward CTTCCTTTGGTTGTCGCTAGTTTGATTGGTGGAGCTG
C11A0 Reverse CAGCTCCACCAATCAAACTAGCGACAACCAAAGGAAG
C308A Forward GGGAAAATATATCCTTGCTTGCCTTTTGGGTCATGCC
C308A Reverse GGCATGACCCAAAAGGCAAGCAAGGATATATTTTCCC
C309A Forward GGGAAAATATATCCTTTGTGCTCTTTTGGGTCATGCC
C309A Reverse GGCATGACCCAAAAGAGCACAAAGGATATATTTTCCC
C363A Forward CCTCTTATGATGAAGGCTGTTGAAGAGAACAATGGTGTC
C363A Reverse GACACCATTGTTCTCTTCAACAGCCTTCATCATAAGAGG
C395A Forward CGCATTGTTTCAAgcaGTCGCTGCCGTTTTCATTGC
C395A Reverse GCAATGAAAACGGCAGCGACtgcTTGAAACAATGCG
C467A Forward GGTTGACAGATCTGCTACCGTCTTGAACG
C467A Reverse CGTTCAAGACGGTAGCAGATCTGTCAACC

### 4.3. Recombinant Production of hASCT2 WT and Mutants

To obtain the recombinant hASCT2-His_6_ proteins, 10 µg of pPICZB-ASCT2-His_6_ WT or mutant constructs were linearized with *Pme*I and used to transform *P. pastoris* wild type strain X-33 by electroporation [24]. Putative multi-copy recombinants were selected using YPDS plates containing 2000 µg/mL Zeocin and analysed after 3 days. For large scale protein production, transformed *P. pastoris* cells were inoculated in BMGY medium and grown at 30 °C. To induce the over-expression of hASCT2 WT and mutants, *P. pastoris* cells were centrifuged to remove the BMGY medium and resuspended at final OD of 1 in 250 mL BMMY medium containing 0.5% of methanol, placed in a 2 L conical flask. The growth in methanol was performed at 30 °C for 3 days adding fresh methanol every 24 h to replace the consumed one. In the case of C363A mutant, the expression was obtained growing cells in BMMY medium at 20 °C for only one day. To obtain the membrane fraction, 30 g of *P. pastoris* cells were resuspended in 400 mL of a buffer containing 50 mM Tris, pH 7.4, 150 mM NaCl, 6 mM β-mercaptoethanol and 0.5 mM PMSF and disrupted using bead beater (BioSpec Product). The bead beater chamber was loaded with the cell suspension mixed with glass beads (0.5 mm). After 5 min almost 90% of cell wall was destructed. The cell suspension was centrifuged at 8000× *g* for 30 min and the supernatant containing membrane and cytosolic fractions was collected. This supernatant was ultracentrifuged in a JA30.50 rotor at 108,000× *g* for 2 h. The resulting membrane pellet was washed with urea buffer (5 mM Tris pH 7.4, 2 mM EDTA, 2 mM EGTA and 4 M urea) and then ultracentrifuged as above. The washed membrane fraction (pellet) was resuspended at a final concentration of about 300 mg/mL in a buffer containing 25 mM Tris, pH 7.4, 250 mM NaCl, 6 mM β-mercaptoethanol and 10% glycerol and homogenized using a potter homogenizer. Aliquots of 3 mL of the membrane fraction were stored at −80 °C.

### 4.4. Solubilization and Purification of hASCT2 WT and Mutants

For large-scale solubilization and purification hASCT2 WT and mutants, about 1.5 g of washed membranes (400 mg/mL) was solubilized using a buffer containing 25 mM Tris, pH 7.4, 250 mM NaCl, 6 mM β-mercaptoethanol, 10% glycerol and 2% C_12_E_8_ (*w*/*w*) by agitation for 3 h at 4 °C. Then, a centrifugation at 120,000× *g* for 1h was performed to recover the solubilized materials which were applied to 3 mL Ni-nitrilotriacetic acid (NTA) agarose resin equilibrated with the equilibration buffer (20 mM Tris pH 7.4, 300 mM NaCl, 10% glycerol, 6 mM β-mercaptoethanol, 0.03% C_12_E_8_, and 50 mM imidazole). This mixture was incubated with gentle agitation at 4 °C over night. The Ni-NTA resin was subsequently packed into a column and washed with 30 mL of the equilibration buffer. Then, 3 mL of the same buffer containing 300 mM imidazole and 4 mL of the same buffer containing 500 mM imidazole (referred as elution buffers) were added. Fractions containing purified protein were pooled to 2.5 mL and desalted on a PD-10 desalting column pre-equilibrated with desalting buffer (20 mM Tris pH 7.4, 100 mM NaCl, 10% glycerol, 6 mM β-mercaptoethanol and 0.03% C_12_E_8_), from which 3.5 mL were collected. The amount of recombinant hASCT2 WT and mutants was estimated from Coomassie blue stained 12% SDS-PAGE gels by using the Chemidoc imaging system equipped with Quantity One software (Bio-Rad) as previously described [25]. For Western Blot analysis, purified hASCT2 was immuno-detected incubating membrane with conjugated anti-His antibody 1:1000 for 1 h at room temperature. The reaction was detected by Electro Chemi Luminescence (ECL) assay in dark room.

### 4.5. Reconstitution of the hASCT2 into Liposomes

The purified hASCT2 WT and mutants were reconstituted by removing the detergent using the batch-wise procedure in which mixed micelles of detergent, protein and phospholipids were incubated with 0.5 g Amberlite XAD-4 resin under rotatory stirring (1200 rev/min) at room temperature (23 °C) for 40 min [26]. The composition of the initial mixture was: 200 µL of the solubilized protein WT or mutants (5 µg protein), 120 µL of 10% C_12_E_8_, 100 µL of 10% egg yolk phospholipids (*w*/*v*) in the form of sonicated liposomes prepared as previously described [27], 10 mM L-Gln, 20 mM Tris/HCl pH 7.0 in a final volume of 700 µL. All the operations were performed at room temperature.

### 4.6. Transport Measurements

To remove the external compounds prior uptake experiments, 600 µL of proteoliposomes was passed through a Sephadex G-75 column (0.7 cm diameter × 15 cm height) pre-equilibrated with 20 mM Tris/HCl pH 7.0 and sucrose at an appropriate concentration to balance the internal osmolarity. Uptake was started, at 25 °C by adding 50 µM [^3^H]Gln and 50 mM Na-gluconate to 100 µL proteoliposomes. Transport reaction was stopped by adding 10 µM HgCl_2_; according to the inhibitor stop method, the same inhibitor was added at time zero to control samples (blank) [28]. At the end of transport, 100 µL of proteoliposomes was passed through a Sephadex G-75 column (0.6 cm diameter × 8 cm height) to separate the external from the internal radioactivity. Liposomes were eluted with 1 mL 50 mM NaCl and collected in 4 mL of scintillation mixture, vortexed and counted. The experimental values were analysed by subtracting to each sample the respective controls (blank); the initial rate of transport was measured by stopping the reaction after 15 min, i.e., within the initial linear range of [^3^H]Gln uptake into the proteoliposomes. Grafit 5.0.13 software was used to calculate kinetic parameters, to derive IC50 values in inhibition assays and to measure transport rate by first order rate equation.

### 4.7. Homology Modelling of hASCT2

The homology structural model of the hASCT2 was built on the basis of the human glutamate transporter EAAT1 (PDB 5LLU) used as template [19]. The alignment was used to run the program Swiss Model [29].

## 5. Conclusions

The present work, for the first time, gains insights into the structure/function relationships of the human ASCT2. Taken together, the above described results indicated that hASCT2 undergoes an ON/OFF regulation due to SH/S-S formation and that this might be due to the different mobility of the elevator portion of the protein across the plasma membrane, as suggested for bacterial homolog GLTph. Moreover, the C467 residue was identified as part of the substrate binding site of hASCT2 being involved in Gln recognition and translocation. Besides deciphering the molecular basis of translocation, these findings can be of great importance also in applied research, such as pharmacology due to the high expression of ASCT2 in several human cancers.

## Abbreviations

C_12_E_8_	Octaethylene glycol monododecyl ether
YPDS	Yeast Extract Peptone Dextrose Sorbitol
BMGY	Buffered Glycerol-complex Medium
BMMY	Buffered Methanol-complex Medium
DTE	DiThioErythritol
MTSES	Sodium (2-sulfonatoethyl)methane thiosulfonate
NEM	*N*-ethylmaleimide
TCA	Tricarboxylic acid cycle
CySS	Cystine
GSH	Reduced Glutathione
GSSG	Oxidized Glutathione
NaHS	Sodium hydrosulfide
GSNO	*S*-nitrosoglutathione
